# MUC16 as a serum-based prognostic indicator of prometastatic gastric cancer

**DOI:** 10.1038/s41598-024-64798-8

**Published:** 2024-07-02

**Authors:** Jieun Lee, Sang Wook Lee, So Hyun Kang, Donghyeok Seol, Mira Yoo, Duyeong Hwang, Eunju Lee, Young Suk Park, Sang-Hoon Ahn, Yun-Suhk Suh, Kyoung Un Park, Nak-Jung Kwon, Hyung-Ho Kim

**Affiliations:** 1 Department of Surgery, Seoul National University Bundang Hospital, 173-82 Gumiro, Bundang-Gu, Seongnam-si, Republic of Korea; 2 Precision Medicine Institute, Macrogen Inc., 254, Beotkkot-ro, Geumcheon-gu, Seoul, Republic of Korea; 3 Department of Surgery, Chung-Ang University Gwangmyeong Hospital, Gwangmyeong-si, Republic of Korea; 4 Department of Laboratory Medicine, Seoul National University Bundang Hospital, Seongnam-si, Republic of Korea; 5 Department of Laboratory Medicine, Seoul National University College of Medicine, Seoul, Republic of Korea; 6 Present Address: Chung-Ang University Gwang Myeong Hospital, Gwangmyeong-si, Republic of Korea; 7 Present Address: Chung-Ang University, College of Medicine, Seoul, Republic of Korea

**Keywords:** Gastric cancer, Metastasis, Serum-based prognostic marker, Olink, MUC16, Cancer, Biomarkers

## Abstract

Metastatic gastric cancer (GC) presents significant clinical challenges due to its poor prognosis and limited treatment options. To address this, we conducted a targeted protein biomarker discovery study to identify markers predictive of metastasis in advanced GC (AGC). Serum samples from 176 AGC patients (T stage 3 or higher) were analyzed using the Olink Proteomics Target panels. Patients were retrospectively categorized into nonmetastatic, metastatic, and recurrence groups, and differential protein expression was assessed. Machine learning and gene set enrichment analysis (GSEA) methods were applied to discover biomarkers and predict prognosis. Four proteins (MUC16, CAIX, 5’-NT, and CD8A) were significantly elevated in metastatic GC patients compared to the control group. Additionally, GSEA indicated that the response to interleukin-4 and hypoxia-related pathways were enriched in metastatic patients. Random forest classification and decision-tree modeling showed that MUC16 could be a predictive marker for metastasis in GC patients. Additionally, ELISA validation confirmed elevated MUC16 levels in metastatic patients. Notably, high MUC16 levels were independently associated with metastatic progression in T3 or higher GC. These findings suggest the potential of MUC16 as a clinically relevant biomarker for identifying GC patients at high risk of metastasis.

## Introduction

Gastric cancer (GC) was the fifth most common malignant tumor in the world and is currently the fourth leading cause of cancer death^1^. Among the possible treatment modalities, such as chemotherapy, immunotherapy, and radiation, surgery is the only curative strategy. The 5-year survival rate for patients at each stage of GC treated with gastrectomy has been reported to be 95.1–88.4% for stage I, 84.0–71.7% for stage II, and 58.4–26.1% for stage III. However, the 5-year survival rate for stage IV GC patients with distant metastasis is approximately 10%, and the survival period is only 14.3–16.6 months^2,3^. Patients with AGC, with a high risk of metastasis and recurrence, have a low survival rate and poor outcomes. Therefore, predicting patients likely to exhibit metastasis and recurrence at an earlier disease stage is very important for treatment outcomes.

Clinically, one of the characteristics of advanced gastric cancer (AGC) with gastric wall invasion of T3 or higher is that the degree of lymph node metastasis and frequency of distant organ metastasis are different even at the same gastric wall invasion depth. Additionally, there is currently no method for predicting the tendency for recurrence even after radical gastrectomy and adjuvant chemotherapy. If we can predict the risk of metastasis or future recurrence of advanced gastric cancer, we can engage in more aggressive treatment at an earlier period of treatment, and even when recurrence is expected but there is no radiological or hematological evidence, we can be more proactive in treatment decision-making.

There are several ongoing studies assessing GC-related antigens as diagnostic biomarkers^4^. Although various methods and techniques have been recommended for ultimately establishing an applicable, sensitive and real-time monitoring system using circulating blood, few methods can currently be applied in clinical practice^5^. Carcinoembryonic antigen (CEA), cancer antigen 19–9 (CA19–9) and cancer antigen 72–4 (CA72–4) are regarded as clinically popular gastrointestinal tumor biomarkers^6^. However, their positivity rates are less than 40%, and the sensitivity and specificity are insufficient. Moreover, they cannot reflect the metastatic status of the tumor initially and have no ability to predict recurrence^7,8^. Therefore, there is an urgent need to identify more precise and effective blood biomarkers to provide optimal management for GC patients. In fact, there is no clear study of a predictive biomarker for GC metastasis and/or recurrence, so continuous efforts are needed to develop diagnostic, prognostic, and therapeutic approaches for these malignancies.

Recently, the focus of protein biomarker research has undergone a paradigm shift from single evaluation of individual biomarkers to integrated analysis of multiple biomarkers for disease diagnosis and prognosis. A prominently employed method in this regard is the antibody-based proximity expansion assay (PEA)^9,10^. Within the PEA framework, each protein within the panel is subject to investigation through a pair of antibody probes, each of which is intricately labeled with oligonucleotides possessing a mutual affinity. This design ensures that in the event both antibodies successfully bind to the target protein in close proximity, the associated oligonucleotides can undergo amplification via DNA polymerase enzymatic activity. As a result, a distinctive DNA sequence is generated, serving as an effective surrogate marker for the respective protein of interest. This unique sequence can subsequently be quantitatively evaluated employing the established technique of quantitative real-time PCR (qPCR)^9^.

This study aimed to identify biomarkers specific for prometastatic GC in the prediction of metastasis and/or recurrence after curative gastrectomy for gastric cancer. We retrospectively analyzed serum protein levels collected on the day of gastrectomy in the group with and without metastasis using the O-link proteomics panel.

## Materials and methods

### Study cohort and serum sample preparation

**Figure 1:**
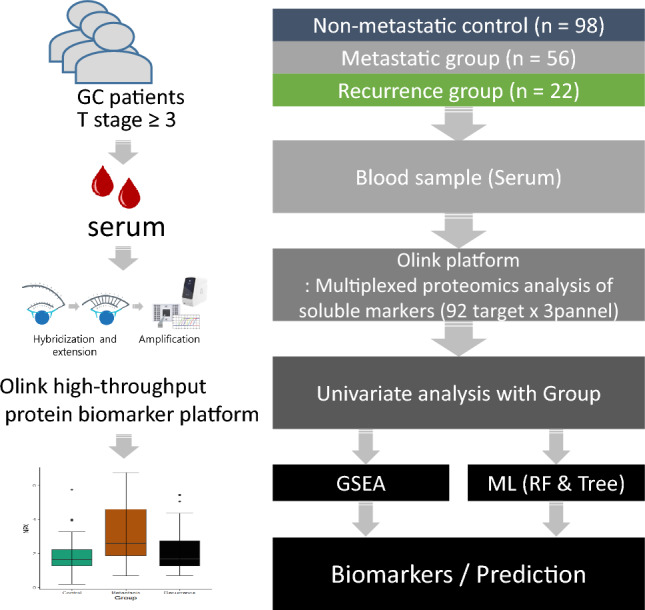
Serum samples collected from 176 patients with gastric cancer were analyzed using an Olink multiplexed high-throughput platform. The study comprised 98 nonmetastatic patients, 56 metastatic patients, and 22 recurrent patients. Machine learning and gene set enrichment analysis methods were applied to discover biomarkers and predict prognosis.

This study included 176 gastric adenocarcinoma patients who underwent gastrectomy at Seoul National University Bundang Hospital (Gyeonggi-do, South Korea) from 2019 to 2022. The key eligibility criteria included (1) pathological diagnosis of gastric adenocarcinoma with available serum samples and follow-up information, (2) eligible serum samples at T stage 3 or higher, (3) no other significant systemic diseases, including autoimmune disorders, and (4) no previous procedures. Serum samples were obtained from 176 retrospectively involved patients. Whole blood was collected in a serum separation tube on the day of gastrectomy. Serum was obtained by centrifuging whole-blood samples at 1500 rpm for 15 min; serum samples were then stored in a deep freezer at − 70 °C until use. The basic clinical characteristics of the patients, including sex and age, are summarized in Table 1. The workflow of the study is shown in Fig. 1. All patients provided informed consent. Samples were procured, and the study was conducted under Institutional Review Board approval before tissue acquisition. Handling and processing of samples was performed according to relevant guidelines and regulations (IRB B-1402–240-004).

**Table 1:** Clinicopathologic features of study cohort.

Group	Control* (N = 98)	Metastasis (N = 56)	Recurrence (N = 22)
Age, median (range)			
	63 (34–87)	56 (24–87)	57 (43–81)
Gender, N (%)			
Male	74 (76%)	26 (46%)	13 (59%)
Female	24 (24%)	30 (54%)	9 (41%)
Lauren’s classification, N (%)			
Intestinal	39 (40%)	1 (2%)	2 (9%)
Diffuse	47 (48%)	13 (23%)	17 (77%)
Mixed	11 (11%)	0	3 (14%)
NA**	1 (1%)	42 (75%)	
Ming’s classfication, N (%)			
Infiltrative	90 (92%)	14 (25%)	21 (95%)
Expanding	7 (7%)	0	1 (5%)
NA**	1 (1%)	42 (75%)	
T stage, N (%)			
T3	53 (54%)	0	2 (95%)
T4a	40 (41%)	11 (20%)	13 (59%)
T4b	5 (5%)	3 (5%)	6 (27%)
NA**	0	42 (75%)	1 (5%)
N stage, N (%)			
N0	29 (30%)	0	3 (14%)
N1 (1–2)	16 (16%)	0	3 (14%)
N2 (3–6)	29 (30%)	2	3 (14%)
N3a (7–15)	24 (245)	1 (2%)	8 (36%)
N3b (> 16)	0	11 (20%)	5 (23%)
NA**	0	42 (75%)	0
M stage, N (%)			
M0	98 (100%)	0	22 (100%)
M1	0	56 (100%)	0
TNM stage, N (%)			
IA, IB	0	0	1 (5%)
IIA, IIB	39 (40%)	0	3 (14%)
IIIA, IIIB, IIIC	59 (60%)	0	18 (82%)
IV	0	56 (100%)	0
Differentiation, N (%)			
Papillary adenocarcinoma	3 (3%)	0	1 (5%)
Well differentiated tubular adenocarcinoma	1 (1%)	0	0
Moderately differentiated tubular adenocarcinoma	29 (30%)	1 (2%)	2 (9%)
Poorly differentiated tubular adenocarcinoma	28 (29%)	3 (5%)	2 (9%)
Mucinous adenocarcinoma	6 (6%)	0	2 (9%)
Poorly cohesive carcinoma	15 (15%)	8 (14%)	8 (36%)
Mixed carcinoma	14 (14%)	2 (4%)	5 (23%)
Other	2 (2%)	42 (75%)	2(9%)
MSI, N (%)			
MSS	80 (82%)	40 (75%)	20 (91%)
MSI-H	9 (9%)	1 (2%)	1 (5%)
MSI-L	8 (8%)	4 (7%)	1 (5%)
NA**	1 (%)	11 (20%)	0
EBV, N (%)			
Negative	93 (95%)	42 (75%)	21 (95%)
Positive	4 (4%)	1 (2%)	1 (5%)
NA	1 (1%)	13 (23%)	0
HER2 IHC, N (%)			
Negative	75 (77%)	38 (68%)	13 (59%)
Positive	22 (22%)	14 (25%)	9 (41%)
NA**	1 (1%)	4 (7%)	0

*Control: T stage 3 or high GC without metastasis and recurrence.

**NA (data not available).

### Serum immune proteomics

Proteomic analyses were performed for all serum samples at the laboratory of Macrogen Precision Medicine Institute (Seoul, South Korea) for measuring circulating protein levels in serum with high sensitivity across a wide range of concentrations^11^ without any other information given. The analyses were based on the Olink Proteomics Target 96 Immuno-Oncology, Target 96 Oncology II and Target 96 Inflammation panels (Olink Proteomics, Uppsala, Sweden) with proximity extension assay (PEA) technology^9,10^, which was based on pairs of antibodies equipped with single-strand oligonucleotide DNA barcodes. Target binding by paired antibodies generated double-stranded DNA amplicons, which were further quantified to indicate protein levels. Analyses were run with the recommended internal control, and interplate variability was adjusted by intensity normalization. Protein levels were given as normalized protein expression (NPX) data, which were relative and log2 transformed. A high NPX value corresponded to a high protein concentration. An increase in the NPX value by 1 corresponded to a doubling of the protein level. The Target 96 Immuno-Oncology, Target 96 Inflammation and Target 96 Oncology II panels included 92 proteins (total 276 proteins; Supplementary Fig. S1) in important immune, inflammation and oncology pathways, as listed in Table S1.

### Data analysis

Relative levels of 279 proteins were reported as NPX units, and the expression distribution of 276 proteins was analyzed using the Benjamini and Hochberg (1995) method for cross/interaction analysis and *p* values. Using a multivariate integrative approach, we identified protein signatures distinguishing metastasis and recurrence samples and attempted to identify clusters or subgroups within patients. Optimization was performed to identify the number of components and features per component using twofold cross-validation and leave-one-group-out-cross-validation, respectively.

The R package “Olink® Analyze” was used for the following analyses. For all 276 protein markers across multiple panels, an analysis of variance (ANOVA) on NPX measurements among patient groups with or without metastasis and recurrence was used to determine significantly differentially expressed protein assays. For adjusted p values, the Benjamini–Hochberg method was applied at a significance threshold of 0.05.

Gene set enrichment analysis (GSEA) was run on the list of all 276 ranked proteins using the gseGO() function of the R package “ClusterProfiler”. All molecular function (MF), biological process (BP), and cellular component (CC) GO terms that were enriched for each comparison, such as metastasis vs. control, metastasis vs. recurrence, and control vs. recurrence, were elevated or suppressed in differentially expressed protein levels with fold changes. The dotplot() and gseaplot2() corresponding to the R package “lattice” (v0.3.1) and “enrichplot” (v.1.20.0) were used to visualize the results of the enriched pathway analysis as a Cleveland dot plot and a regular GSEA plot.

In addition, a random forest model^12^ was trained for predictions on a differentially expressed set of 154 samples from the metastasis group (n = 98) and the control group (n = 56), excluding the 23 patients with recurrence. The R package “randomForest” (v.4.7–1.1) was used to train the random forest model with 500 tree sets for an optimal model. The trained model was assessed to predict how significantly differentially expressed proteins were divided from the output of decision trees by taking each split, and each protein predictor was fitted to feature importance using the mean decrease in the Gini index. The random forest model was evaluated with the area under the curve by using the R package “ROCR” (v.1.0–11) to visualize the performance of the random forest classifications on each protein predictor.

The decision tree ^13^ classified the groups of the metastasis and control samples by segmenting each protein predictor using the R packages “partykit” (v.1.2–16) and “rpart” (v.4.1.19), and the outcome tree was visualized to be interpreted easily. The highest predictive protein markers were listed at the top of the decision tree model, and this conducted the variance importance rankings in the random forest model.

### TCGA public data analysis

mRNA expression data and clinical information of TCGA stomach adenocarcinoma (TCGA-STAD; stad_tcga_pan_can_atlas_2018) were obtained through cBioPortal (https://www.cbioportal.org/)^14^. Patients were divided into three groups according to their expression levels of MUC16: high (top 25%), medium (between the top 25% and top 75%), and low (below the top 75%). Subsequently, Kaplan‒Meier survival analysis was conducted for each TNM stage using the R package “survival” (v.3.5.7).

### Enzyme-linked immunosorbent assay (ELISA)

The levels of human carbohydrate antigen 125 (CA125) in GC were determined using a CA125 ELISA kit (CUSABIO, Houston, TX) according to the manufacturer’s instructions. Briefly, 50 µl of serum was added to a 96-well plate coated with human CA125 antibody. Then, 50 µl of HRP-conjugated mixed solution was added to each well, and the plate was incubated for 1 h at 37 °C. After several washes, the color reaction was developed with the substrate solution and blocked with the stop solution. The optical densities were measured at 450 nm. Statistical analyses were performed using Prism 9.0 (GraphPad Software Inc., San Diego, CA, USA).

### Ethical approval and consent to participate

The study was endorsed by the ethical review committees of Seoul National University Bundang Hospital (IRB no. B-1402-240-004).

## Results

### Cohort population

Metastasis and recurrence are important factors that decrease survival time in AGC. If metastasis or recurrence can be predicted, more aggressive treatment for GC is possible. To discover protein markers that can predict metastasis or recurrence at the serum level, 176 serum samples from patients with T stage 3 or higher disease were collected and divided into 3 groups. The recurrence group included cases where the same type of tumor reappeared in the same area or in another organ after treatment, as indicated by clinical follow-up records. The metastasis group included cases where tumors had developed in other organs at the time of surgery, based on clinical information. The control is a group without metastasis or recurrence. This study included patients with T stage 3 or higher GC without metastasis and recurrence (Control, n = 98), metastatic GC (Meta, n = 56), and recurrence serum from patients with recurrent GC (Recur, n = 22) (Fig. 1). Table 1 shows the clinicopathological characteristics of the study cohorts. In the control group, the intestinal and diffuse types were 40% and 48%, respectively. In the metastasis group, Lauren’s classification could not be identified in 75% of cases, and the diffuse type was identified in 77% of cases in the recurrence group. TNM stage II and III were found in 40% and 60% of cases in the control group, respectively, but stage III was found in 82% of cases in the recurrence group. All metastasis group cases were classified as stage IV. Moderately differentiated tubular adenocarcinoma (30%) and poorly differentiated tubular adenocarcinoma (29%) were most frequently observed in the control group, and poorly cohesive carcinoma (36%) and mixed carcinoma (23%) were frequently observed in the relapse group. MSS type, EBV-negative and HER-2-negative were most common in all three groups.

### Serum-based proteomic analysis

**Figure 2:**
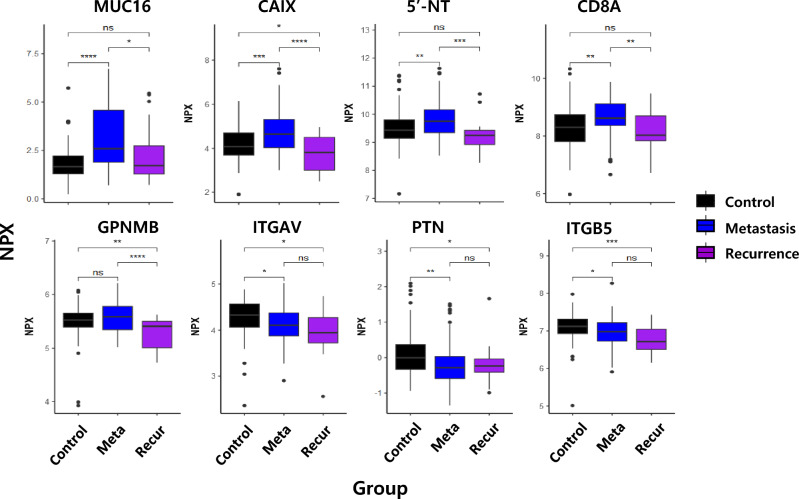
NPX comparison with eight statistically significant proteins. The control group refers to nonmetastatic patients. The largest difference in NPX values between the Control and Meta groups is shown with the Mucin-16 (MUC16) protein assay. Statistically significant differences are denoted against groups. The threshold of the nonparametric analysis of variance method was an adjusted *P* value of < 0.05.

In this study, we analyzed the levels of 276 marker proteins in key immune, oncology and inflammation pathways by PEA using the Olink Target 96 Immuno-Oncology, Target 96 Oncology II and Target 96 Inflammation panels (Supplementary Fig. S1). Three proteins are included in all three panels. A list of proteins used in each panel is shown in Supplementary Table S1. Comparison of protein levels in each group showed the dynamics of serum proteomics. Eight out of 279 proteins showed a significant change in each group (Fig. 2). Among them, Mucin-16 (MUC16), carbonic anhydrase 9 (CAIX), 5’-nucleotidase (5’NT) and T-cell surface glycoprotein CD8 alpha chain (CD8A) were significantly elevated in metastatic GC patients compared with the control subjects. Additionally, MUC16, CAIX, 5’-NT, CD8A, and GPNMB proteins were significantly decreased in the recurrence group compared with the metastatic group. Interestingly, when stratified by sex, MUC16 showed a significant increase only in the female metastatic patients (Supplementary Fig. S2).

### Gene set enrichment analysis (GSEA)

**Figure 3:**
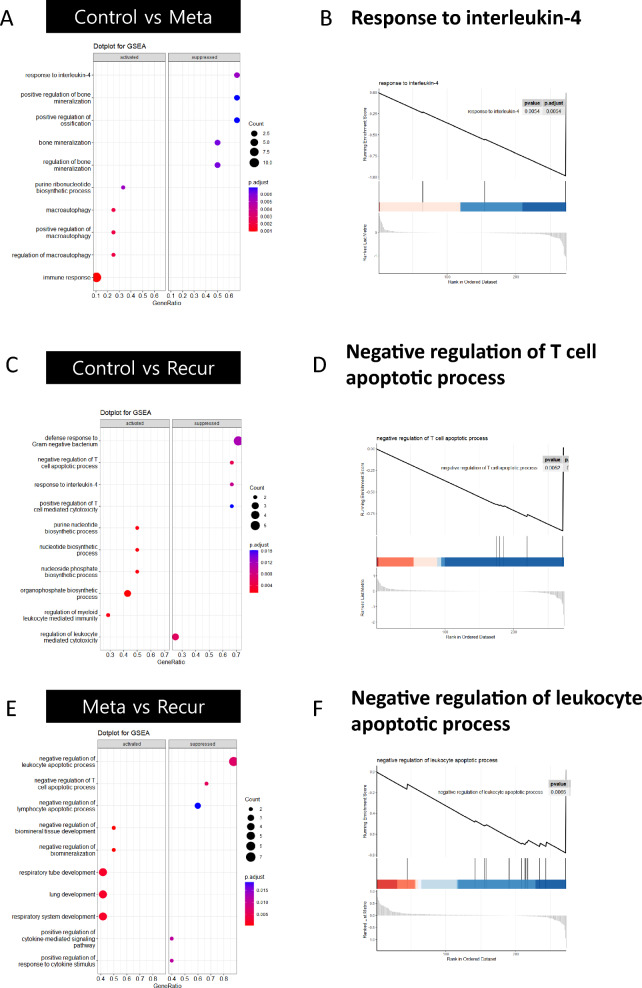
Significantly enriched Gene Ontology (GO) terms. Dot plots and enrichment plots based on GSEA results show GO biological processes on the vertical line, and the area of the circle indicates gene counts. The P value is represented with the depth of colors. The right and left panels denoted as activated and suppressed indicate that represented groups have more activated pathways with higher protein expression levels. (**A**), (**B**) Comparison between Control and Meta groups with the enriched pathway ‘Response to interleukin-4’. (**C**), (**D**) Comparison between Control and Recur groups with the enriched pathway ‘Negative regulation of T-cell apoptotic process’. (**E**), (**F**) Comparison between Meta and Recur groups with the enriched pathway ‘Negative regulation of leukocyte apoptotic process’. R packages were used to generate the plots.

Gene set enrichment analysis (GSEA) showed that the hallmark pathways were altered in the metastasis and recurrence groups compared with the control group (Fig. 3). The functional meaning of upregulated MUC16 comprises activated and suppressed significant alteration pathways such as the negative regulation of the T cell apoptotic process and the response to interleukin-4 in the Metastasis group associated with cancer cell proliferation and malignant phenotypes^15^, such as migration and invasion. At the same time, GSEA demonstrated that the Control group was remarkably enriched in regulating myeloid leukocyte-mediated immunity. Additionally, the cellular response to decreased oxygen levels and hypoxia-related pathways were also enriched in the metastasis group compared with the control group (Fig. 3A, B and Supplementary Fig. S3A). Moreover, the negative regulation of the T-cell apoptotic process and the regulation of leukocyte-mediated cytotoxicity were significantly enriched in the Recurrence group, whereas the Control group was enriched in the organophosphate biosynthetic process and the regulation of myeloid leukocyte mediated immunity (Fig. 3C, D and Supplementary Fig. S3B). These results collectively represent a notable difference in leukocyte immunity between groups. In addition, leukocyte- or lymphocyte-related processes were observed in the recurrence group compared to the metastasis group; on the other hand, the tissue development pathway was increased in the metastasis group compared to the recurrence group (Fig. 3E, F and Supplementary Fig. S3C). Our findings of identified biological pathways demonstrated that the Metastasis and Recurrence groups with a higher expression of MUC16, the more significant discrepancy of the Metastasis group, compared to the Control group showed that cancer development played an important role in metastasis and that innate immunity played an important role in recurrence.

### A random forest classification model

**Figure 4:**
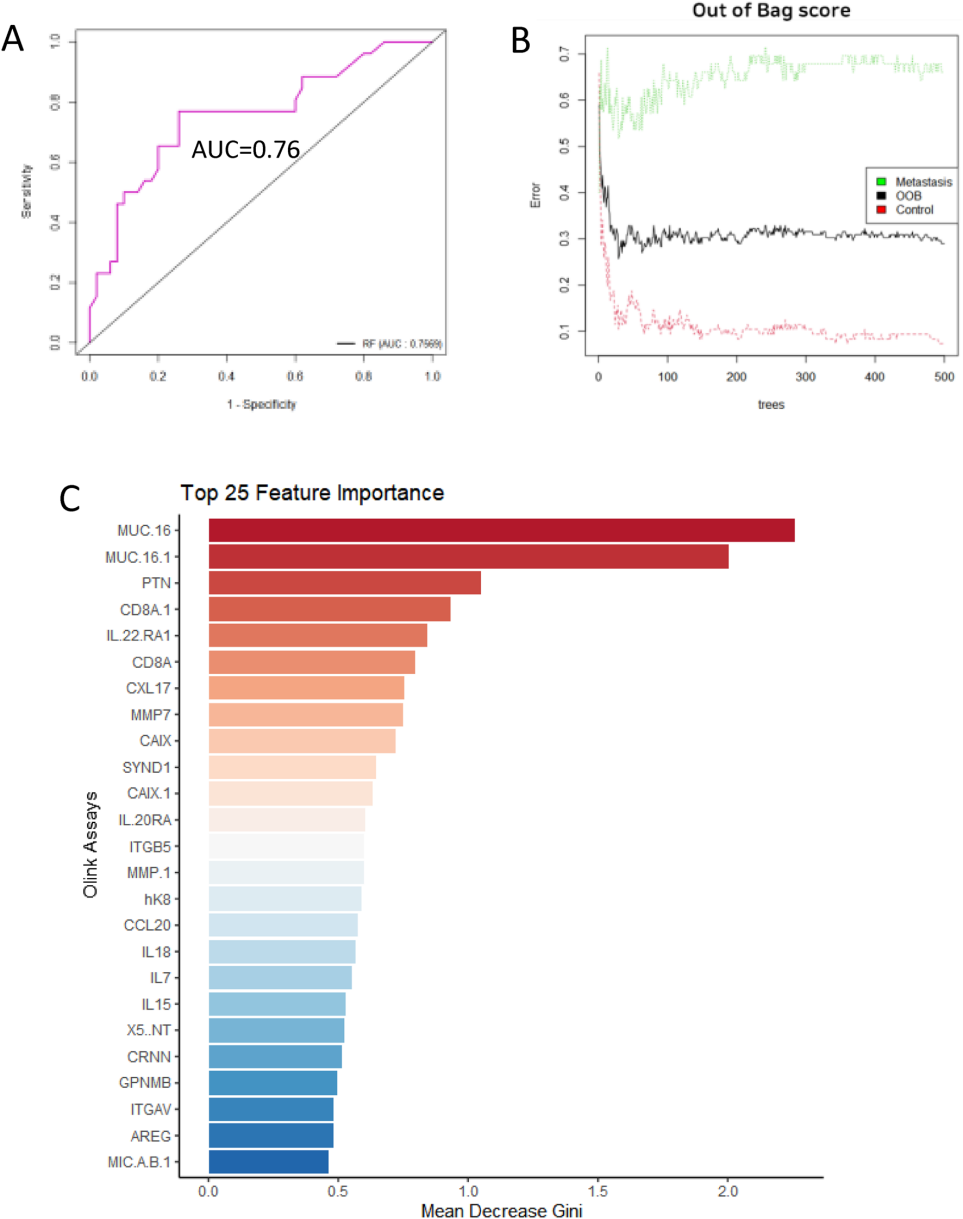
Importance of random forest models. (**A**) Evaluation of RF using the area under the curve measure. (**B**) Out-of-bag score means the error rate when random forest models are established. (**C**) Bar plot of variable importance of the top 25 protein assays is shown.

To evaluate the random forest model, we estimated an area under the curve (AUC) of 0.76 for the discovery cohort, and this information is illustrated in Fig. 4A. The out-of-bag (OOB) error of the established random forest model was calculated based on the nonselected samples from the metastasis and control groups on the iteration of the random forest algorithm. This information is illustrated in Fig. 4B. A rapid decrease in the OOB error estimation was observed from the beginning to the 30th tree, which was the number of trees used in the model. The number of trees was optimal at 500 in our random forest model since the OOB score was stable after 500. The overall OOB was approximately 29% when the number of trees was 500.

A random forest classification model using the 500 trees built on the training and test datasets of the metastasis and control groups resulted in the most predictive potential for MUC16. The protein assays are listed in descending order with the score of the mean decrease Gini. This represented the importance of the feature, while the small values were barely influential in the discovery model. Out of the 276 total protein assays, MUC16 was clearly the most useful (mean decrease Gini > 2.0) for predicting prometastatic gastric cancer as plotted with the highest mean decrease Gini (Fig. 4C). The Immuno-Oncology and Oncology II panels had identical MUC16 protein assays that led to duplicated names in the plot. Although two different panels were used at different time points, both MUC16 assays from these panels indicated that MUC16 was the most important biomarker predictor in the random forest model. This model was visualized as a scaled boxplot for the 25 proteins according to the result of the feature importance.

The diagram of the decision-tree model for the metastasis and control samples is illustrated in Supplementary Fig. S4. The decision tree method was used for classification since the performance was better than that of other methods^16^. It was shown as a tree structure with three types of nodes, a decision node, a leaf node and an internal node, and was developed with the nonparametric algorithm for 276 protein assays in 154 patients. The final branches at the bottom of the tree represented the output through the selection. MUC16 also dominated this decision tree model, as proposed in the other analyses.

### MUC16 as a prometastatic prediction marker in GC serum

CEA and CA19–9 were demonstrated to be prognostic factors for gastric cancer^17,18^. Supplementary Table S2 shows the preoperative CEA and CA19-9 levels in 176 patients. Although the measured CEA values increased during metastasis or relapse, they were within the normal range (0 ~ 5) on average, and approximately 17% of each group had values out of the normal range (> 5). Moreover, the average CA19-9 level was significantly increased in approximately 25% of metastatic patients, and 33% of the recurrence group was out of the normal range (> 37).

**Figure 5:**
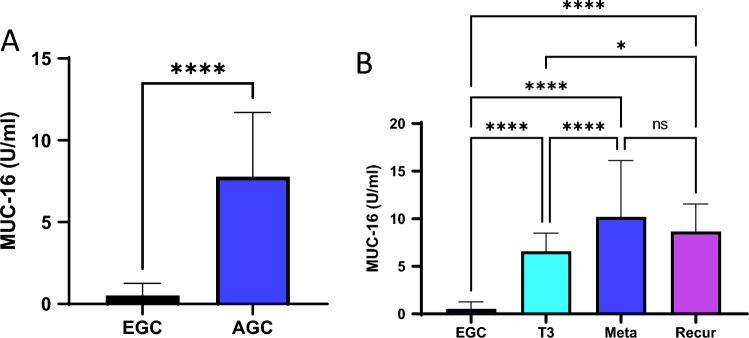
Evaluation of the prognostic value of the MUC16 level in GC serum. (**A**) The expression level of MUC16 in serum samples from EGC (n = 46) and AGC (n = 246) patients was measured with ELISA. (**B**) MUC16 unit increased in the metastasis group (n = 69) and the recurrence group (n = 22) compared to the AGC group (n = 155) without metastasis and recurrence. *P* values less than 0.05 are given one asterisk, and *P* values less than 0.0001 are given four asterisks. ns means *P* > 0.05.

We further evaluated the prognostic value of the MUC16 level in the GC serum samples. First, the expression level of MUC16 (CA125) in the serum of EGC and AGC patients was measured with ELISA. The MUC16 level was increased by 15-fold in the AGC samples compared to the EGC samples (Fig. 5A). Additionally, the MUC16 unit was increased by 20-fold in the metastasis group and by 16-fold in the recurrence group compared to the EGC group (Fig. 5B). In the TCGA stomach cancer tissue data, the mRNA expression level of MUC16 was significantly increased according to stage (Supplementary Fig. S5A). However, there was no significant difference in survival rates in terms of MUC16 expression at each stage in the TCGA data (Supplementary Fig. S5B).

## Discussion

Biopsy procedures, which involve invasive surgical interventions to obtain tissue samples from the affected site, have traditionally been the primary approach for diagnosing cancer. Unfortunately, these procedures are often painful for patients and can be challenging, especially in cases where cancer has spread to multiple sites. Additionally, the need for repeated biopsies can place a substantial burden on patients^19^. As a result, there is a growing interest in exploring noninvasive alternatives, such as blood sample-based diagnostics, for the detection of cancer.

A recent study demonstrated the stratification of plasma samples from most cancer types with high sensitivity and specificity and the detection of patients with early disease, as exemplified by early-stage patients with lung and colorectal cancers^20^. However, although they used plasma samples from 1477 cancer patients, they did not include blood samples from patients with stomach cancer. Here, we analyzed the levels of 276 marker proteins related to immune, oncology, and inflammation pathways in serum samples from GC patients using O-link panels. Four proteins were found to be common to all three panels, and a total of nine proteins exhibited significant changes in different groups, including metastatic and recurrence patients.

In particular, we suggest that MUC16 may serve as a predictive marker for metastasis in GC patients. MUC16, also known as cancer antigen 125 (CA-125), is a large glycoprotein found on the surface of various cancer cells, including GC. It has been extensively researched as a potential biomarker for different types of cancers, such as ovarian, pancreatic, and bladder cancer^21^. Importantly, the levels of MUC16 in blood serum are regularly monitored in ovarian cancer patients, and an increase in its concentration from an individualized baseline level is considered a prognostic indicator of cancer recurrence^22^. Studies have demonstrated that MUC16 mutations are also associated with increased cancer cell growth and metastasis^23,24^. Moreover, its overexpression has been linked to poorer prognosis in various types of malignancies^25^. Here, we found that high expression levels of MUC16 in GC patient serum samples were an independent predictor of metastatic progression in T3 or higher GC. Additionally, MUC16 expression levels in cancer tissues were significantly increased with increasing stage in TCGA stomach cancer public data (Supplementary Fig. S5). Huang L. et al. analyzed the MUC16 mutational signature using cBioPortal data and found no difference in MUC16 mRNA expression between GC samples with wild-type MUC16 compared with mutated MUC16, but overall survival revealed that patients with MUC16 mutation had longer survival^26^.

The CAIX (carbonic anhydrase IX) protein is often associated with cancer, particularly with solid tumors^27^. CAIX is an enzyme that plays a role in regulating pH levels in cells and is overexpressed in various cancer types. CAIX is overexpressed in response to hypoxic conditions in various cancer types^28^. Its expression is associated with aggressive tumor behavior and poorer outcomes, making it a target of interest for both diagnostic and potential therapeutic interventions in cancer. Interestingly, the serum level of CAIX was higher in the metastasis group than in the control group.

5’-NT (5’-nucleotidase, also known as CD73) is an enzyme that plays a significant role in cancer, particularly in the context of the tumor microenvironment and immune response^29^. 5’-Nucleotidase (CD73) protein expression in cancer is closely linked to the tumor microenvironment and immune response, and high levels of CD73 can promote an immunosuppressive environment, allowing cancer cells to grow and metastasize^29^. It is a potential target for cancer immunotherapies, and its expression can serve as a diagnostic and prognostic marker for certain cancer types. Interestingly, the serum level of 5’-NT was higher in the metastatic GC group than in the control group.

CEA is a glycoprotein often elevated in the blood of individuals with various cancers, including gastric cancer. Elevated carbohydrate antigen CA19-9 levels in gastric cancer patients are often associated with more advanced disease stages and may indicate a less favorable prognosis^30^. However, it is important to note that these markers lack specificity for gastric cancer and can be influenced by various factors^6^, so they are typically used in conjunction with other clinical and diagnostic information. Here, we used GSEA to identify characteristic pathways associated with metastasis and recurrence. Specific pathways, such as the response to interleukin-4 and hypoxia-related pathways, were enriched in metastatic patients (Fig. 3). In contrast, the negative regulation of T-cell apoptosis and nucleotide metabolic processes was downregulated in the recurrence group, indicating differences in the underlying mechanisms. We used a random forest classification model to evaluate the predictability of various proteins. A decision-tree model was also used for classification and demonstrated that MUC16 played a dominant role in the model (Fig. 4). This means that this model is useful for predicting GC metastasis based on the MUC16 serum level in AGC patients. In fact, MUC16 is a protein that has shown promise as a novel biomarker in cancer, particularly in the context of ovarian cancer^31^. In this study, we found the potential of MUC16 as a novel marker for predicting metastasis in gastric cancer and underscored its superiority compared to traditional markers such as CEA and CA19-9. However, it also acknowledges that further research is needed to fully understand the clinical implications of these findings, particularly regarding survival rates.

Our study has several limitations. The exploratory nature of our investigation precludes immediate clinical implementation of the results. It is worth noting that our AGC cohort is drawn from a relatively homogeneous Korean population, which may limit the extrapolation of our findings to more diverse populations. However, we are not aware of any specific population characteristics that would significantly impact the generalizability of our results. It is important to acknowledge that our study population was primarily composed of T stage 3 patients, with only 25.9% having stage III disease. Consequently, our findings may be primarily influenced by the protein levels in patients with the highest tumor burden, resulting in more pronounced protein level variations. Additionally, our results are limited by the preselection of proteins by Olink, and proteins found in other studies of patients with AGC were not examined. Above all, the clinical application of prognostic protein indices hinges on comprehensive validation. If validated, these prognostic indices could serve as a valuable tool for guiding clinical decisions in collaboration with the patient.

### Supplementary Information

Supplementary Information 1.

Supplementary Information 2.

## Data Availability

All data generated or analyzed during this study are included in this published article (and its supplementary information files).

## Abbreviations

GC: Gastric cancer
AGC: Advanced gastric cancer
EGC: Early gastric cancer
GSEA: Gene set enrichment analysis
PEA: Proximity expansion assay
ELISA: Enzyme-linked immunosorbent assay
MUC16: Mucin-16
CAIX: Carbonic anhydrase 9
5’-NT: 5’-Nucleotidase (CD73)
CD8A: T-cell surface glycoprotein CD8 alpha chain
CEA: Carcinoembryonic antigen
CA19-9: Cancer antigen 19–9
