# Supplementary material for: MUC16 as a serum-based prognostic indicator of prometastatic gastric cancer
Source: Sci Rep. 2024 Jul 2;14:15173. doi: 10.1038/s41598-024-64798-8 (PMC11220052; doi:10.1038/s41598-024-64798-8)
Supplement: Supplementary file 1 — Supplementary Information 1. [file 41598_2024_64798_MOESM1_ESM.pptx]

## Slide 1
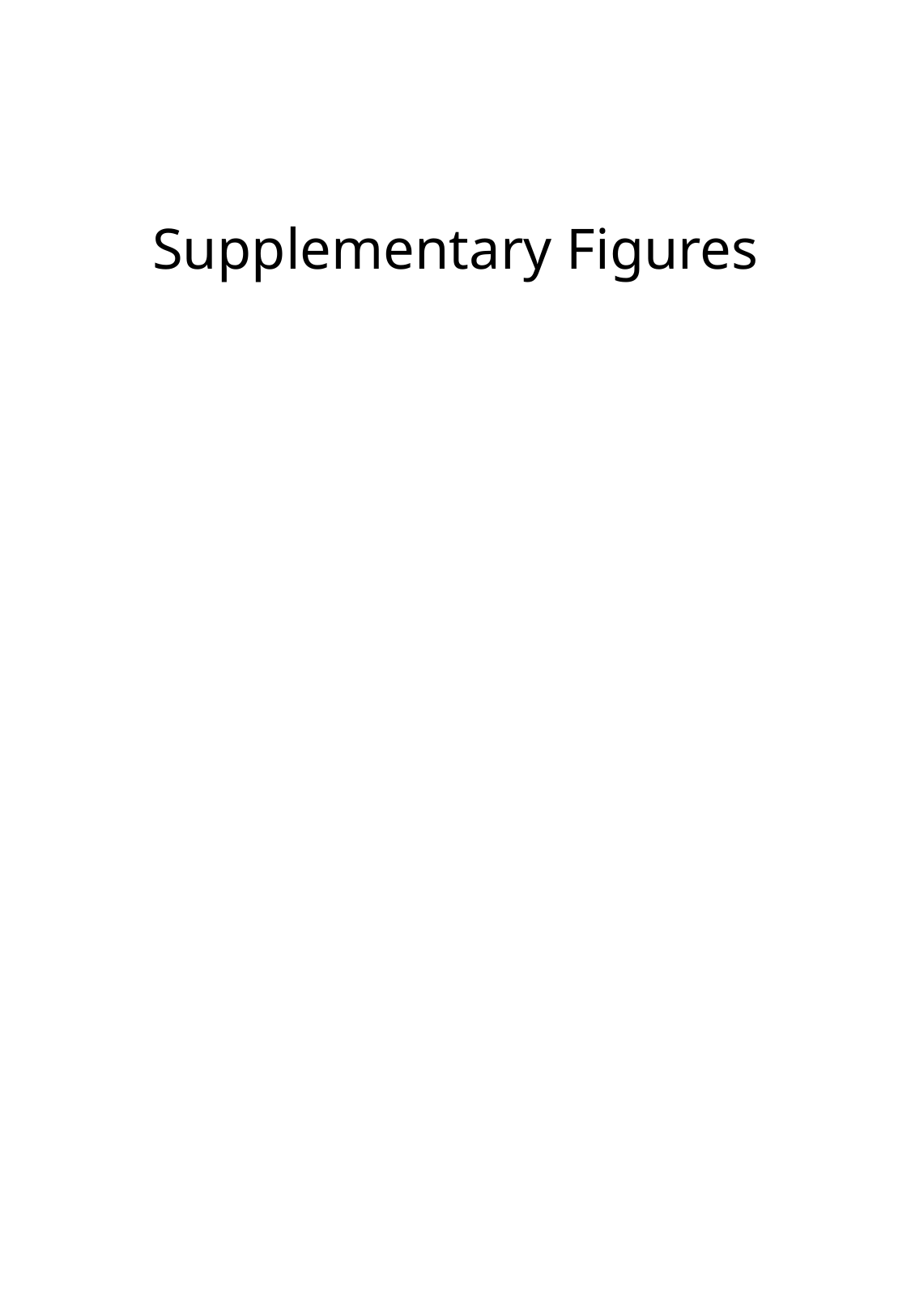

Supplementary Figures

## Slide 2
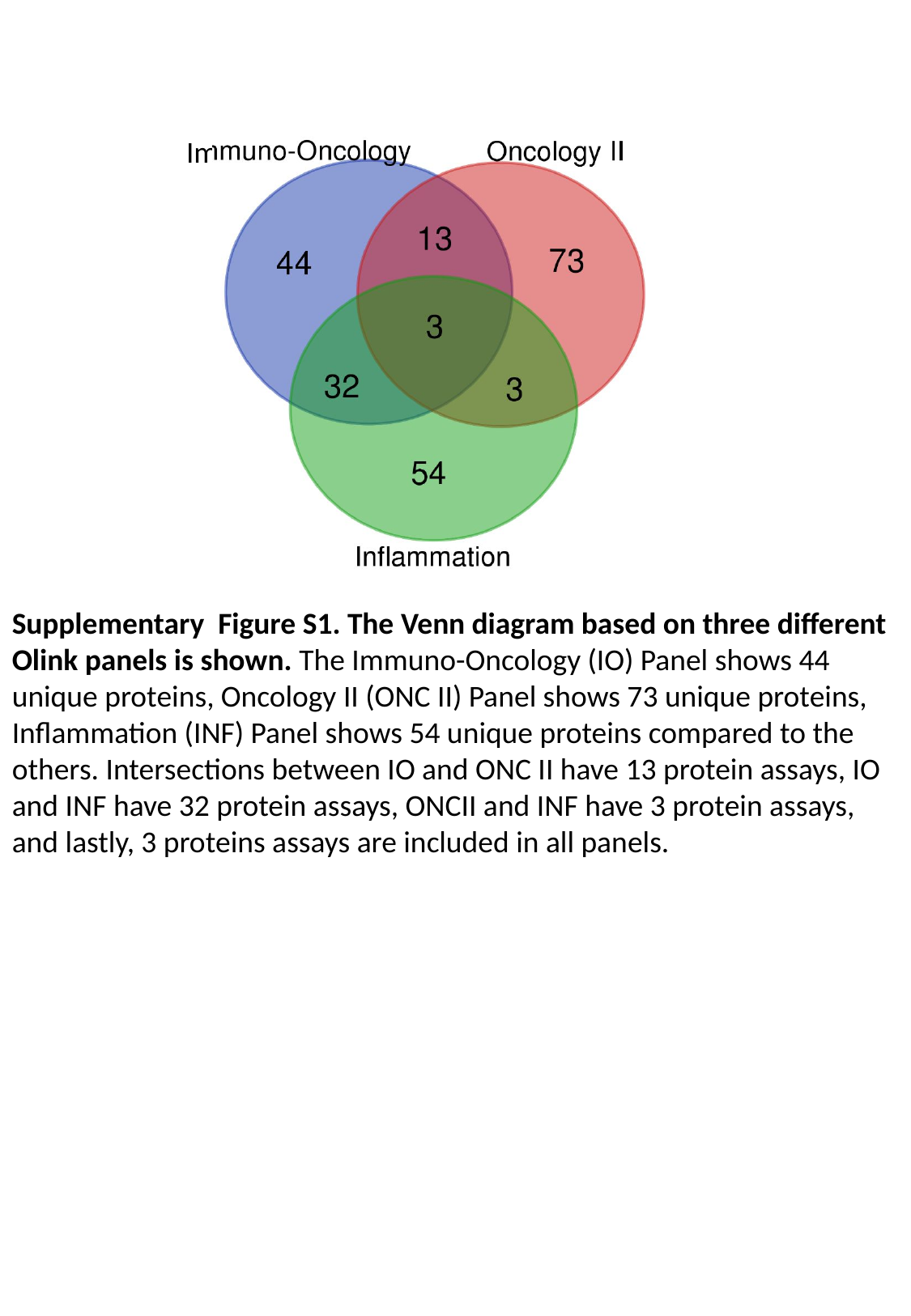

Im
Supplementary Figure S1. The Venn diagram based on three different Olink panels is shown. The Immuno-Oncology (IO) Panel shows 44 unique proteins, Oncology II (ONC II) Panel shows 73 unique proteins, Inflammation (INF) Panel shows 54 unique proteins compared to the others. Intersections between IO and ONC II have 13 protein assays, IO and INF have 32 protein assays, ONCII and INF have 3 protein assays, and lastly, 3 proteins assays are included in all panels.

## Slide 3
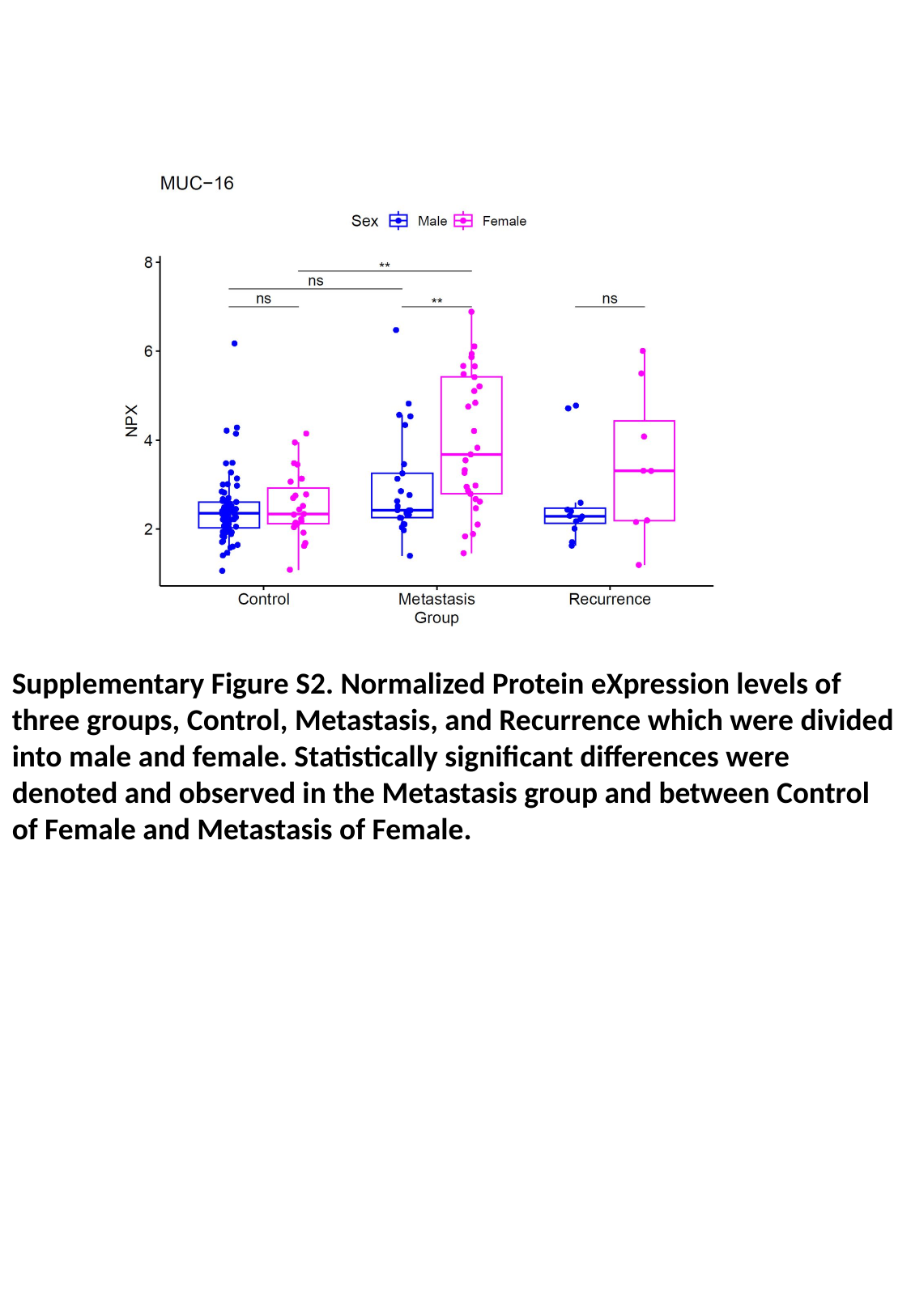

Supplementary Figure S2. Normalized Protein eXpression levels of three groups, Control, Metastasis, and Recurrence which were divided into male and female. Statistically significant differences were denoted and observed in the Metastasis group and between Control of Female and Metastasis of Female.

## Slide 4
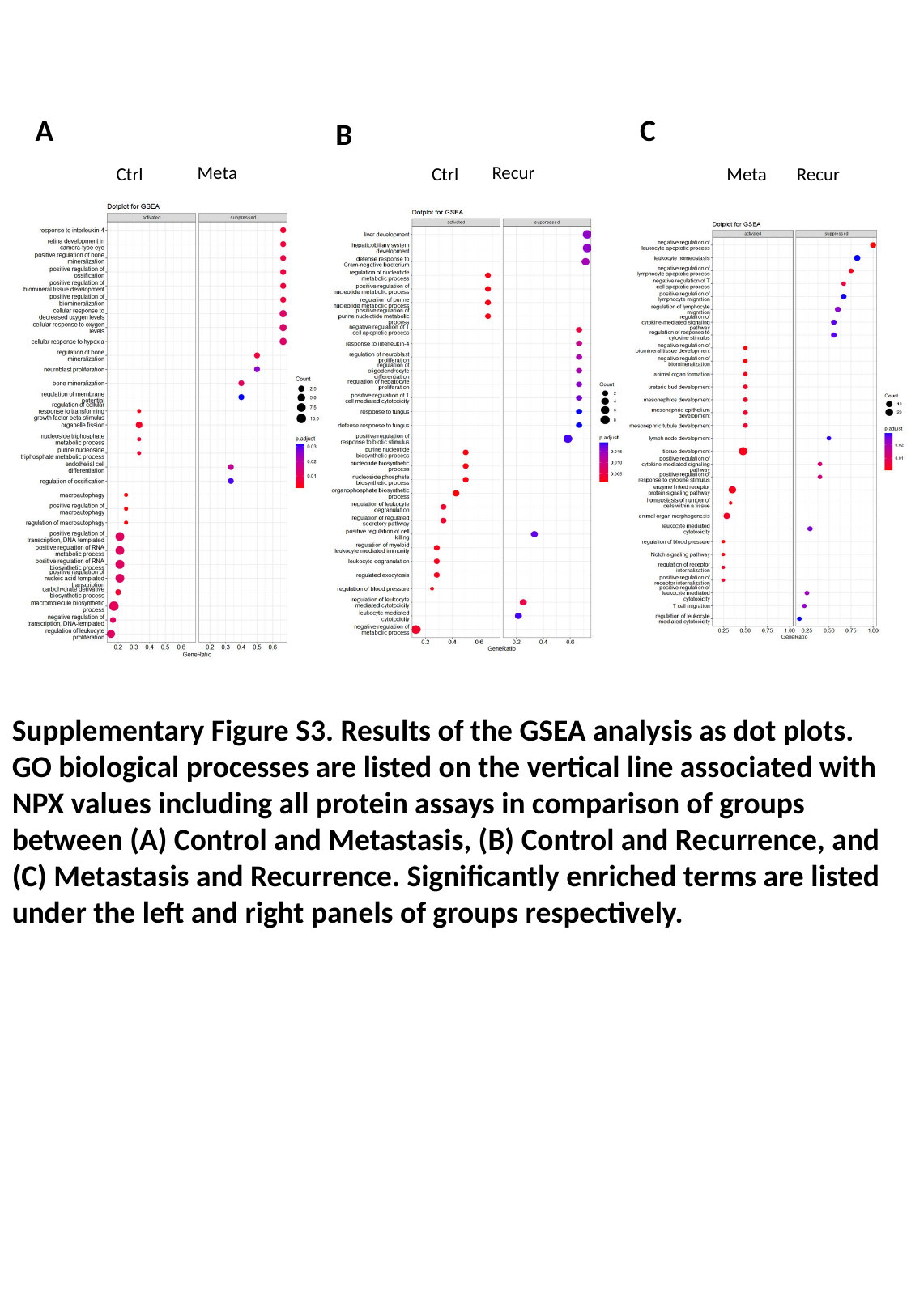

A
C
B
Meta
Recur
Ctrl
Ctrl
Meta
Recur
Supplementary Figure S3. Results of the GSEA analysis as dot plots. GO biological processes are listed on the vertical line associated with NPX values including all protein assays in comparison of groups between (A) Control and Metastasis, (B) Control and Recurrence, and (C) Metastasis and Recurrence. Significantly enriched terms are listed under the left and right panels of groups respectively.

## Slide 5
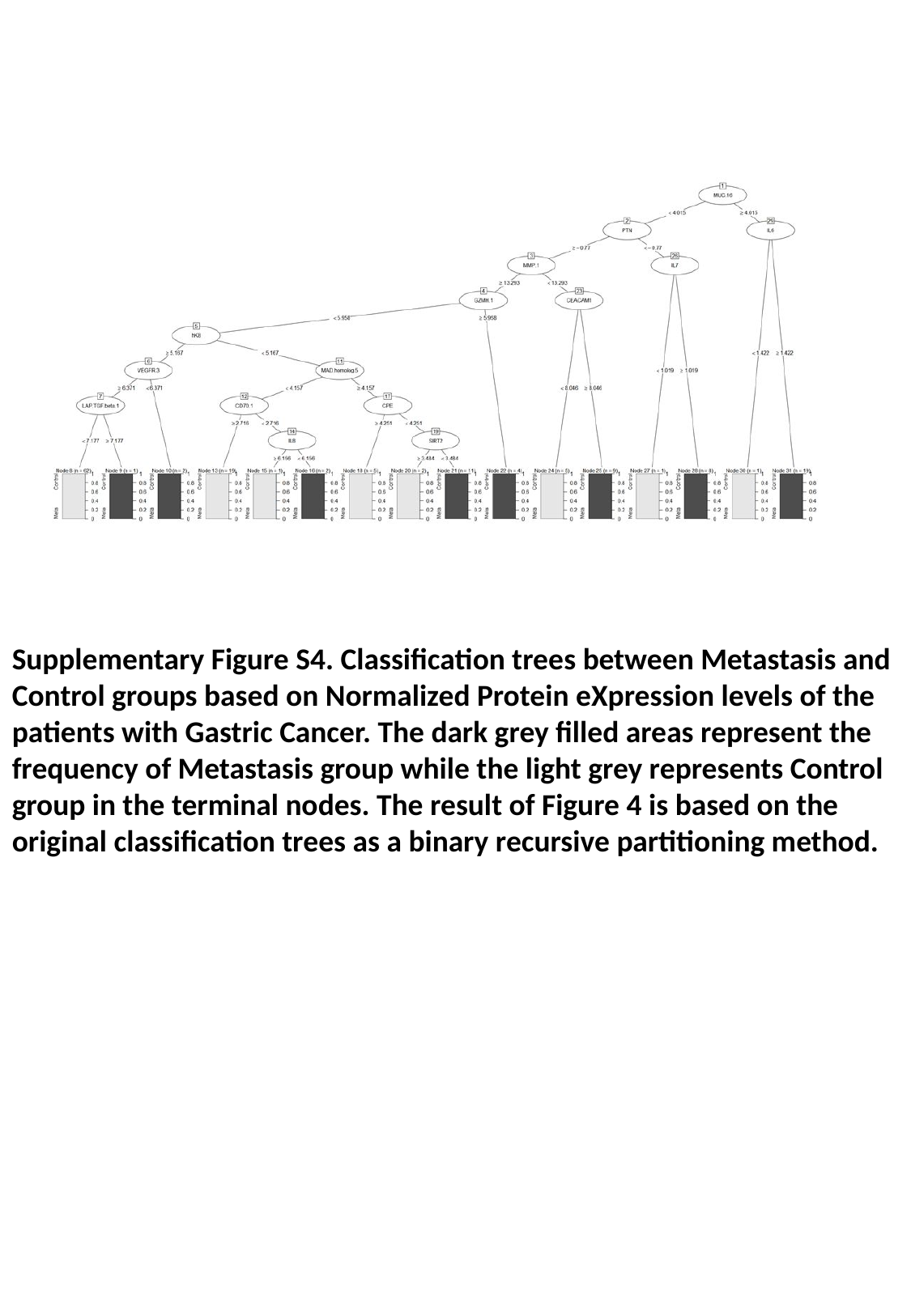

Supplementary Figure S4. Classification trees between Metastasis and Control groups based on Normalized Protein eXpression levels of the patients with Gastric Cancer. The dark grey filled areas represent the frequency of Metastasis group while the light grey represents Control group in the terminal nodes. The result of Figure 4 is based on the original classification trees as a binary recursive partitioning method.

## Slide 6
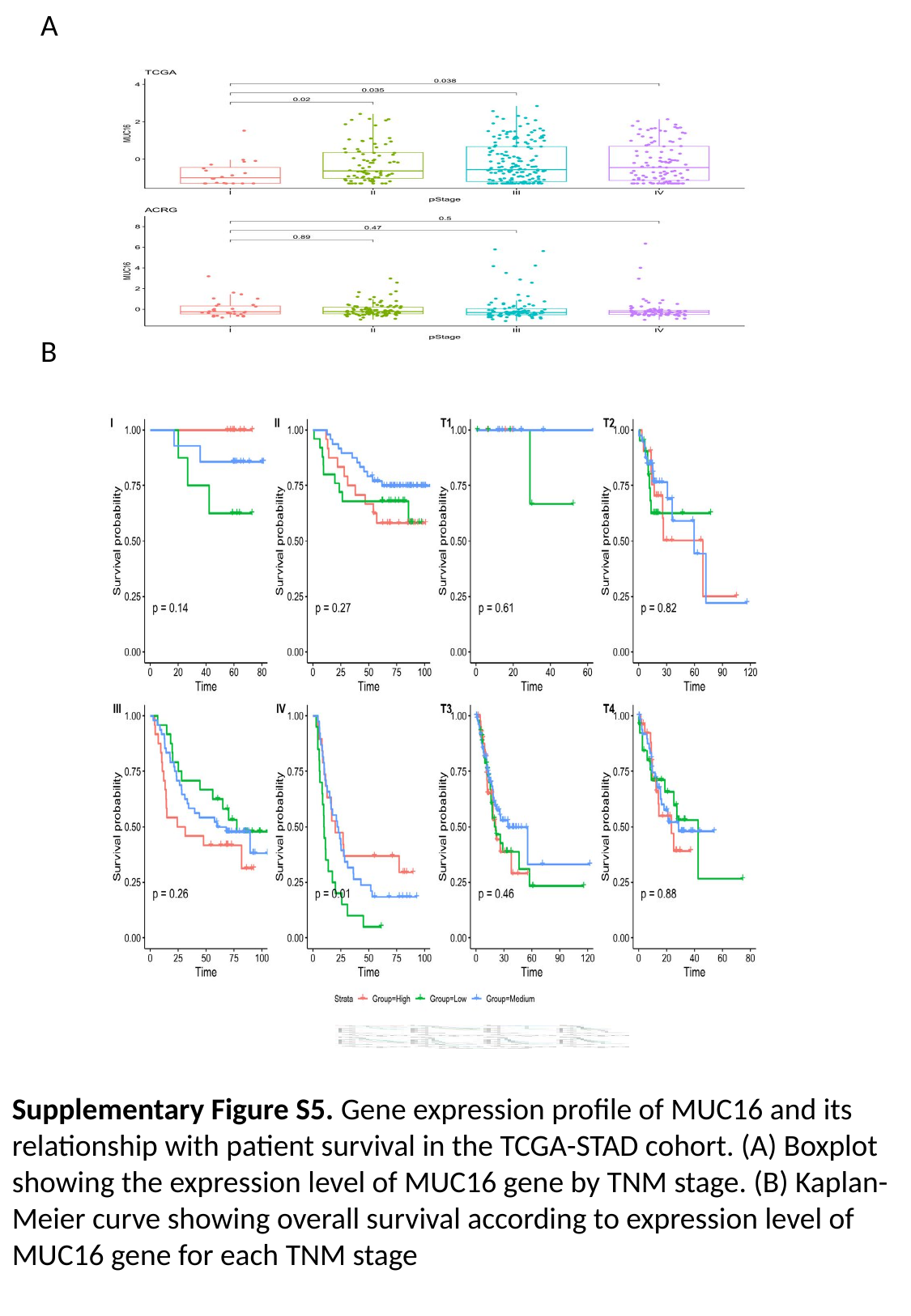

A
B
Supplementary Figure S5. Gene expression profile of MUC16 and its relationship with patient survival in the TCGA-STAD cohort. (A) Boxplot showing the expression level of MUC16 gene by TNM stage. (B) Kaplan-Meier curve showing overall survival according to expression level of MUC16 gene for each TNM stage
